# Childhood trauma and suicide risk in hospitalized patients with schizophrenia: the sequential mediating roles of pandemic related post-traumatic stress symptoms, sleep quality, and psychological distress

**DOI:** 10.3389/fpsyt.2023.1221529

**Published:** 2023-09-21

**Authors:** Min Xie, Xuemin Zou, Yingjing Xie, Li Hu, Yiguo Tang, Jai Cai, Yunxue Kuang, Ling Zhu, Min Zou, Qiang Wang

**Affiliations:** ^1^Mental Health Center, West China Hospital, Sichuan University, Chengdu, Sichuan, China; ^2^Sichuan Clinical Medical Research Center for Mental Disorders, Chengdu, China; ^3^The First People’s Hospital of Longquanyi District Chengdu, West China Longquan Hospital Sichuan University, Chengdu, China; ^4^Department of Clinical Psychology, Southwest Hospital, The First Hospital Affiliated to Army Medical University (Third Military Medical University), Chongqing, China; ^5^Department of Pharmacy, West China Hospital, Sichuan University, Chengdu, China

**Keywords:** COVID-19 pandemic, post-traumatic stress symptoms, schizophrenia, childhood trauma, sleep quality, suicide risk

## Abstract

**Introduction:**

Stressful global situation due to the COVID-19 pandemic caused a tremendous impact on mental health in hospitalized patients with schizophrenia. The mediating roles of psychological impact related to COVID-19, sleep quality, and psychological distress were investigated in the association between childhood trauma and suicidal risk in hospitalized patients with schizophrenia.

**Methods:**

We analyzed cross-sectional data of 147 patients with schizophrenia and 189 healthy controls (HCs).

**Results:**

Histories of childhood trauma and schizophrenia were good predictors of COVID-19-related psychological impact, global sleep quality, and psychological distress. Moreover, the series mediation model showed that the effect of childhood trauma on suicidal risk in hospitalized patients with schizophrenia was totally sequential mediated by the psychological impact of COVID-19, sleep quality, and psychological distress.

**Conclusion:**

Clinicians need to recognize the increased suicidal risk associated with COVID-19-related psychological distress in schizophrenia patients with a history of childhood trauma.

## 1. Introduction

Schizophrenia is a severe mental disorder with an excess of early mortality rate (1). And suicide is a leading cause of premature death (2). A meta-analysis of 26 studies showed the lifetime and point prevalence of suicidal ideation were 34.5% (95% CI, 28.2–40.9%), and 29.9% (95% CI, 24.2–35.6%) in schizophrenia, respectively (3). Another meta-analysis of 81 studies found that patients with schizophrenia who reported suicidal ideation had a 5.8-fold higher risk for future suicide than those without suicidal ideation (4). Suicide attempts are more or less likely to happen in patients with schizophrenia due to their specific psychological vulnerabilities combined with prevailing social norms and economic factors. Previous studies found that males, younger age, financial stressors, unemployment, alcohol consumption, childhood trauma, isolation, loneliness, having previously attempted suicide or family histories of suicide, and a pandemic are predictive of increased suicide risk (5–8). Meanwhile, public health responses to those risk factors may mitigate suicide risk associated with the novel coronavirus disease 19 (COVID-19) pandemic (9).

Since the outbreak of COVID-19 spread around the world, the shift in the focus of the health system and the pandemic prevention policies have brought dramatic changes in people’s lives, including social isolation, unemployment, and excessive COVID-19 media exposure (10–12). Measures taken to prevent the spread of COVID-19 may have a particularly high psychological impact on patients with schizophrenia (13). Previous studies confirmed that psychosocial stress, depression, and anxiety related to COVID-19 were very common in the population, especially in those patients with mental illness (14–18). One study found that patients with nonaffective psychosis had experienced more psychological impacts related to COVID-19 than healthy controls (HCs) (19). Another case-report study found that environmental risk factors, such as the COVID-19 pandemic, may trigger relapse in patients with schizophrenia (20). This suggests that effective psychological support for schizophrenia during the COVID-19 pandemic may need to be given more attention. Moreover, a review summarized that there were broad impacts of COVID-19 on psychiatric patients, including exacerbated psychosis, depression, and suicidal ideation, which suggested that a transformation of psychiatric care is essential during the pandemic (21).

Childhood trauma, including abuse and neglect during early childhood, was associated with a higher risk, persistent symptoms and increased suicidal risk in patients with schizophrenia (8, 22). A previous study found that childhood trauma increased the risk of admixtures of four major mental symptom domains (affective, psychotic, anxiety, and manic psychopathology), whether in a representative general population, mood disorders, anxiety disorder or schizophrenia (23). To date, several theoretical models could explain the potential pathway between childhood trauma and suicide. The stress sensitization hypothesis suggests that individuals who have experienced childhood trauma are at a higher risk of developing psychiatric problems when they encounter stressful life events, compared to those who have not experienced childhood trauma (24). Furthermore, the stress-diathesis model describes a collection of suicide-related traits that influence the probability of engaging in suicidal behavior in response to stressors. These traits are associated with various combinations of abnormalities in neural circuitry and neurotransmitter systems (25).

Since the outbreak of COVID-19 in late 2019, individuals with childhood trauma have had more psychological impact, depression, anxiety, and stress than those without a childhood trauma history (26). John-Henderson found that psychological impacts related to COVID-19 mediated the association between childhood trauma and sleep quality (27). Laskemoen et al. found that insomnia symptoms played a mediating role in the relationship between childhood trauma and the severity of depressive and anxiety symptoms in patients with severe mental illness (28). In addition, a study of a chain mediation model showed the perceived impact of the pandemic, and global sleep quality were sequential mediators between childhood trauma (predictor) and consequent mental health status (outcome) in the general population (26). Further, childhood trauma was associated with increased suicidal risk in schizophrenia (8, 29). There was supportive evidence that the association between childhood trauma and suicide was mediated by sleep quality, perceived stress, or hyperarousal symptoms (30–32).

To date, few studies have investigated the relationships among childhood trauma, pandemic-related post-traumatic stress disorder (PTSD) symptoms, global sleep quality, psychological distress, and suicidal risk during the COVID-19 pandemic in patients with schizophrenia. Thus, in this study, we aimed to (1) compare childhood trauma, psychological impact related to COVID-19, global sleep quality and mental health outcome between schizophrenia patients and HCs; (2) examine whether childhood trauma and history of schizophrenia were risk factors for psychological impact, global sleep quality, and mental health outcomes of depressive, anxiety and stress in all participants; and (3) explore whether there were mediating effects of traumatic symptoms related to COVID-19, global sleep quality and psychological distress between childhood trauma and suicidal risk in hospitalized patients with schizophrenia.

## 2. Methods

### 2.1. Design

In a cross-sectional study conducted from August 2021 to July 2022, a total of 336 participants participated in a web-based survey. Patients with schizophrenia were recruited from inpatient ward of West China Hospital, Mental Health Center. Age- and sex-matched healthy controls were recruited from WeChat Moments. All participants’ data were collected using electronic questionnaires via the online survey tool Wen-juan-xing.^1^ All patients completed the questionnaire within 1 week of hospitalization, specifically during their acute episodes. Additionally, we verified the completion of the questionnaire on the same day as its submission. Primary inclusion criteria for all the participants were aged from 16 to 55 years old and able to use smartphones and complete questionnaires. Patients with schizophrenia were diagnosed with ICD-10 by psychiatrists. Invalid and incomplete questionnaires were excluded. Healthy controls were excluded who had been diagnosed with mental illness by psychiatrists according to self-reported questionnaires (Have you been diagnosed with a mental illness by a psychiatrist?). All subjects included in the analysis voluntarily participated in the survey and completed the questionnaires.

### 2.2. Data collection

#### 2.2.1. Demographic variables

The questionnaires included general information (age, sex, height, weight, education level, marital status and income level), mother’s and father’s education levels. The education level includes (1) illiteracy; (2) primary school; (3) middle school; (4): vocational high school; (5) senior high school; (6) junior college; (7) bachelor’s degree; (8) master’s degree; and (9) and doctor’s degree or PhD. Married status included single, married and divorced or other. The income levels were self-reported incomes compared with their local people, which were divided into 5 categories: (1) Low-income level; (2) Low-Middle-income level; (3) Middle-income level; (4) Middle-High-income level; and (5) High-income level.

#### 2.2.2. Childhood trauma questionnaires

Childhood trauma experiences before 16 years old were collected using Childhood Trauma Questionnaire-Short Form (CTQ-SF), including emotional abuse, physical abuse, sexual abuse, emotional neglect and physical neglect (33). A 5-point Likert scale (1 = not at all, 2 = occasional, 3 = sometimes, 4 = often, and 5 = very often) was used to assess the trauma severity related to certain events or situations during childhood. The CTQ-SF consists of 25 clinical items and 3 validity items. The 25 clinical items incorporated 5 dimensions, including emotional abuse, physical abuse, sexual abuse, emotional neglect and physical neglect, and ranged from 5 to 25 for each subscale. The cutoffs of each subscale were used to define which subjects experienced any subtype of childhood trauma: (1) emotional abuse ≥13, (2) physical abuse ≥10, (3) sexual abuse ≥7, (4) emotional neglect ≥15, and (5) physical neglect ≥10. Based on these cut-off scores, we defined individuals who had experienced childhood trauma as having experienced one or more of subtype childhood trauma. The CTQ total score was the sum score of 5 subscales, ranging from 25 to 125, which indicated the severity of traumatic experiences during childhood. The Chinese version of the CTQ-SF has good reliability and validity among hospitalized patients with schizophrenia in China (34).

#### 2.2.3. Impact of event scale-revised

Psychological impacts related to the COVID-19 epidemic in all participants were collected using impact of event scale-revised (IES-R), which is a self-administered questionnaire. The IES-R has 22 items that incorporate 3 subscales, including intrusion, avoidance, and hyperarousal. The Chinese version of the IES-R showed good reliability and validity for the assessment of PTSD symptoms severity in the Chinese population (35).

#### 2.2.4. The Pittsburgh sleep quality index questionnaire

The Pittsburgh sleep quality index (PSQI) is a self-rated questionnaire used to assess sleep quality and disturbances during the last month. The sum scores of seven components, including subjective self-reported sleep quality, sleep latency, sleep duration, habitual sleep efficiency, sleep disturbances, usage of sleeping medication, and daytime dysfunction, yield one score of global sleep quality, which ranged from 0 to 21. A cutoff value of 5 for the total PSQI score could better distinguish good sleepers and poor sleepers (36).

#### 2.2.5. Depression, anxiety, and stress scale

The short-form version of the depression, anxiety, and stress scale (DASS-21) was used to measure psychological distress, including depression, anxiety, and stress in the last week. The DASS-21 subscales were divided into normal (0–7), mild (8–9), moderate (10–14), severe (15–19), and extremely severe (>19) for anxiety; normal (0–9), mild (10–13), moderate (14–20), severe (21–27), and extremely severe (>27) for depression; and normal (0–14), mild (15–18), moderate (19–25), severe (26–33), and extremely severe (>33) for stress (19). The Chinese version of the DASS-21 for the total scale demonstrated good internal consistency, with a Cronbach’s alpha of 0.95 in the Chinese population (37).

#### 2.2.6. The nurses’ global assessment of suicide risk

Nurses’ global assessment of suicide risk (NGASR) is a useful template for the nursing assessment of suicide risk (38), which is used to access the suicidal risk of schizophrenia in our study. NGASR scale contains 15 items with different weightings, ranging from 0 to 25. The NGASR was divided into low (0–5), moderate (5–8), high (8–11), and extremely high (12+) risk of suicide.

### 2.3. Data analysis

Quantitative variables were tested for normal distribution using the Shapiro–Wilk test. All variables were not normally distributed. The Mann–Whitney U test was used for quantitative variable comparisons between schizophrenia patients and HCs. Pearson’s chi-square test was used for categorical variable comparisons between groups. Then, in all participants, we conducted a hierarchical linear regression to estimate the roles of a history of schizophrenia and childhood trauma on psychological distress, psychological impact related to COVID-19 (scoring of the IES-R), and global sleep quality (scoring of the PSQI) by sequentially adding predictors into 3 blocks within each model. The variables were added into models via the three steps: Step 1: inputted the demographic characteristics of age, sex, BMI, and educational level; Step 2: incorporated socioeconomic status (income level and educational level of parents) into the model, but the additional variance explanation for outcomes was not significant, thus excluding the socioeconomic factors from covariates, so we only added group into Step 2; Step 3: added total score of CTQ. Spearman correlation analysis was carried out to calculate correlation coefficients between total scale scores of the CTQ, the perceived psychological impact of the COVID-19 pandemic, and psychological distress in patients with schizophrenia. Finally, chain mediation analyses were used to examine the mediated effects of the perceived impact of the COVID-19 pandemic and global sleep quality on the relationship between childhood trauma and psychological distress in schizophrenia [i.e., childhood trauma (X), total score of IES-R (M1), total score of PSQI (M2) and DASS-21 (M3) and NGASR total score (Y)]. The chain mediation analyses were conducted by Process 3.5 for SPSS version 24.0 (Model 6). The significance levels of direct, indirect, and mediated effects were set as two-tailed *p* < 0.05. All continuous variables were standardized and then included in regression and mediation analyses performed in SPSS 24.0.

## 3. Results

**Figure 1:**
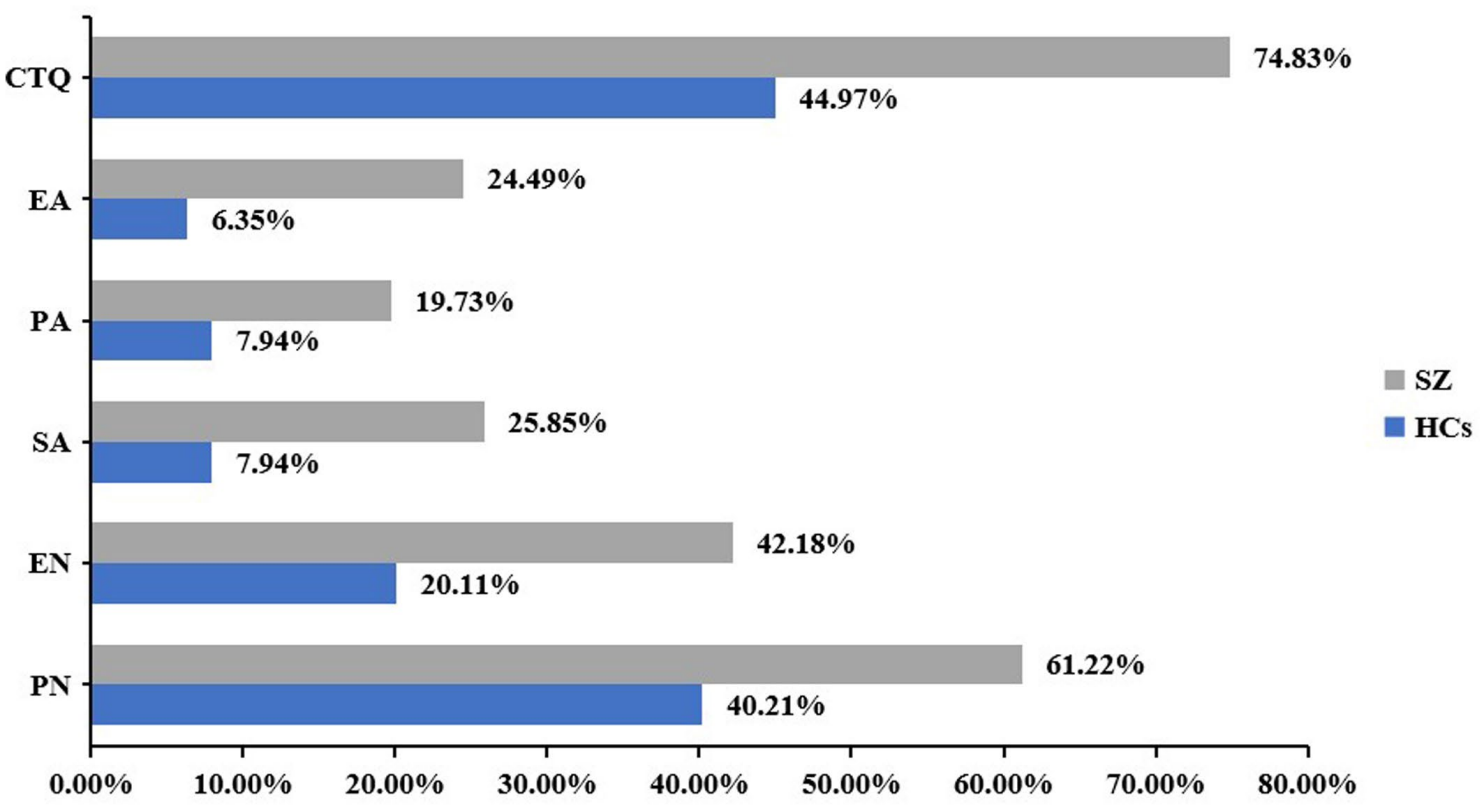
Percentages of history of childhood trauma and subtypes trauma in schizophrenia and healthy controls. CTQ, childhood trauma questionnaire; EA, emotional abuse; EN, emotional neglect; PA, physical abuse; PN, physical neglect; SA, sexual abuse.

A total of 336 participants, of whom 147 were schizophrenia patients and 189 were HCs, were consecutively included. The mean ages of schizophrenia patients and HCs were 28.05 ± 8.77 and 26.78 ± 8.24, respectively. The differences in the educational level of the participants and their parents, income level, and smoking status were not significant between groups (all *p* > 0.05). The BMI of schizophrenia patients was significantly higher than that of HCs (*p* < 0.001). The percentage of single, divorced or separated status in schizophrenia patients was higher than that in HCs, and the percentage of married schizophrenia patients was lower than that in HCs (*χ*^2^ = 25.949, *p* < 0.001). The total scale and subscales (emotional abuse, physical neglect, sexual abuse, emotional neglect, and physical neglect) of the CTQ of schizophrenia were obviously higher than those of HCs (*p* < 0.001) (Table 1). Hospitalized patients with schizophrenia comparisons endorsed significantly more childhood trauma overall (*χ*^2^ = 30.264, *p* < 0.0001), as well as significantly more experiences of the various trauma subtypes compared to HCs (all *p* < 0.001; Figure 1).

**Table 1:** Sociodemographic characteristics of the patients and the control group.

	SZ (*n* = 147)	HCs (*n* = 189)	Test statistic	*p*
Age	28.05 ± 8.77	26.78 ± 8.24	*z* = 0.1120	0.263
Gender (M/F)	65/82	91/98	*χ*^2^ = 0.514	0.474
BMI	23.95 ± 4.59	21.86 ± 3.56	*z* = 4.258	< 0.001
Smoking, n (%)	20 (13.61)	28 (14.81)	*χ*^2^ = 0.099	0.753
EDU level	5.14 ± 1.63	5.66 ± 1.47	*z* = 2.987	0.003
Mother EDU level	3.54 ± 1.69	3.25 ± 1.49	*z* = 1.378	0.168
Father EDU level	3.82 ± 1.73	3.65 ± 1.54	*z* = 0.815	0.415
Income level	2.44 ± 0.95	2.39 ± 0.83	*z* = 0.407	0.684
Marital status, n (%)			*χ*^2^ = 25.949	< 0.001
Single	104 (70.75)	101 (53.44)		
Married	26 (17.69)	80 (42.33)		
Divorced, separated	17 (11.56)	8 (4.28)		
Childhood trauma questionnaire (CTQ)				
Mean CTQ score	47.33 ± 14.73	37.88 ± 12.55	*z* = 6.476	<0.001
EA	9.65 ± 4.35	7.01 ± 3.24	*z* = 6.322	<0.001
PA	7.30 ± 3.36	6.26 ± 2.98	*z* = 4.074	<0.001
SA	6.82 ± 2.94	5.62 ± 2.37	*z* = 6.114	<0.001
EN	13.03 ± 5.73	9.96 ± 5.01	*z* = 5.072	<0.001
PN	10.54 ± 3.57	9.02 ± 3.66	*z* = 4.127	<0.001

BMI, body mass index; CTQ, childhood trauma questionnaires; EA, emotional abuse; EDU, education; EN, emotional neglect; PA, physical abuse; PN, physical neglect; SA, sex abuse.

The mean scores of the DASS-21 total scale and subscales of anxiety, depression and stress in schizophrenia patients were significantly higher than those in HCs (*p* < 0.001). In schizophrenia, 32.66, 19.4, and 16.32% reported severe to extremely severe anxiety, depressive and stress symptoms, respectively. In HCs, 4.77, 3.71, and 1.58% reported severe to extremely severe anxiety, depressive and stress symptoms, respectively. The mean scores of the IES-R total scale and subscales of avoidance, intrusion and hyperarousal in schizophrenia patients were obviously higher than those in HCs (*p* < 0.001). The total PSQI score in schizophrenia patients was higher than that in HCs (*p* < 0.001). There were 56.52 and 30.69% poor sleep in schizophrenia patients and HCs, respectively. And there were 22.76% of schizophrenia reported a high to extremely high risk of suicide (Table 2).

**Table 2:** DASS-21, IES-R, PSQI scores and suicidal risk of participants.

	SZ	HCs	Test statistic	*p*
Depression, anxiety, stress and stress scale-21 (DASS-21)				
Mean DASS-21 total score	18.76 ± 14.87	7.05 ± 9.60	*z* = 8.265	<0.001
DASS-21 (anxiety)	12.10 ± 9.79	4.01 ± 5.95	*z* = 8.727	<0.001
No (0–7)	53 (36.05)	150 (79.37)	*χ*^2^ = 72.939	<0.001
Mild (8–9)	11 (7.48)	9 (4.76)		
Moderate (10–14)	35 (23.81)	21 (11.11)		
Severe (15–19)	20 (13.61)	5 (2.65)		
Extremely severe (20+)	28 (19.05)	4 (2.12)		
Mean DASS-21 depression score	11.77 ± 10.89	5.06 ± 7.33	*z* = 6.743	<0.001
No (0–9)	68 (46.26)	164 (77.25)	*χ*^2^ = 41.390	<0.001
Mild (10–13)	20 (13.61)	20 (10.58)		
Moderate (14–20)	31 (21.09)	16 (8.47)		
Severe (21–27)	11 (7.48)	2 (1.06)		
Extremely Severe (28+)	17 (11.56)	5 (2.65)		
Mean DASS-21 Stress score	13.65 ± 10.64	5.04 ± 6.90	*z* = 8.214	<0.001
No (0–14)	94 (63.95)	176 (93.12)	*χ*^2^ = 46.151	<0.001
Mild (15–18)	15 (10.20)	6 (3.17)		
Moderate (19–25)	14 (9.52)	4 (2.12)		
Severe (26–33)	14 (9.52)	2 (1.06)		
Extremely severe (34+)	10 (6.8)	1 (0.53)		
Impact of event scale-revised (IES-R)				
Mean IES-R score	20.49 ± 16.83	9.21 ± 11.50	*z* = 6.740	<0.001
Mean IES-R avoidance score	7.50 ± 6.68	3.26 ± 4.59	*z* = 6.291	<0.001
Mean IES-R intrusion score	6.16 ± 6.34	3.50 ± 4.55	*z* = 3.742	<0.001
Mean IES-R hyperarousal score	6.8 ± 5.71	2.44 ± 3.60	*z* = 7.916	<0.001
Pittsburgh sleep quality index (PSQI), *n* = 327				
PSQI Global Sleep Quality score	6.53 ± 4.13	4.07 ± 2.89	*z* = 5.636	<0.001
Good sleep quality (0–5)	60 (40.48)	131 (69.31)	*χ*^2^ = 21.913	<0.001
Poor sleep quality (6-21)	78 (56.52)	58 (30.69)		
Nurses global assessment of suicide risk (NGASR), *n* = 145				
Low-risk (0–5)	65 (44.83)	–	–	–
Moderate-risk (5–8)	47 (32.41)	–	–	–
High-risk (8–11)	25 (17.24)	–	–	–
Extremely high-risk (12+)	8 (5.52)	–	–	–

DASS-21, Depression, Anxiety, Stress and Stress Scale-21; IES-R, Impact of Event Scale-Revised; NGASR, Nurses global assessment of suicide risk; PSQI, Pittsburgh sleep quality index.

The results of hierarchical linear regression analysis showed that histories of childhood trauma and schizophrenia were significant predictors of COVID-19-related psychological impact, psychological distress and PSQI global sleep quality (all *p* < 0.001), with adjustment for age, sex, BMI and education level. In Step 1, the covariates explained 5.60, 4.50, and 5.60% of the total variance in the IES-R, DASS-21, and PSQI total scale scores, respectively. In Step 2, when we added group into the model, the additional variance explained 11.6, 16.3, and 11.3% of the total variance in IES-R (∆*R*^2^ = 0.116, *F* = 45.974, *p* < 0.001), DASS-21 (∆*R*^2^ = 0.163, *F* = 67.113, *p* < 0.001) and PSQI (∆*R*^2^ = 0.113, *F* = 45.138, *p* < 0.001) total scale scores. In the final regression model (model 3), when we added the total CTQ score, the additional variance explained 6.8, 7.1, and 3.3% of the total variance in the IES-R (∆*R*^2^ = 0.068, *F* = 29.179, *p* < 0.001), DASS-21 (∆*R*^2^ = 0.071, *F* = 32.292, *p* < 0.001) and PSQI (∆*R*^2^ = 0.033, *F* = 12.906, *p* < 0.001) total scale scores, respectively (Table 3).

**Table 3:** Hierarchical multiple linear regression analyses between history of schizophrenia, CTQ total scale score and DASS-21, IES-R, and PSQI global sleep quality scores in all participants.

	IES-R			DASS-21			PSQI global sleep quality		
Step 1	*R*^2^ = 0.056, *F* = 4.832, *p* = 0.001			*R*^2^ = 0.045, *F* = 3.897, *p* = 0.004			*R*^2^ = 0.056, *F* = 4.756, *p* = 0.001		
	*β*	SE	*p*	*β*	SE	*p*	*β*	SE	*p*
Age	−0.034	0.056	0.547	−0.026	0.056	0.641	0.140	0.056	0.013
Sex	0.327	0.113	0.004	0.272	0.113	0.017	0.225	0.114	0.050
BMI	0.192	0.056	0.001	0.196	0.056	0.001	0.050	0.057	0.373
Education	−0.047	0.035	0.178	−0.006	0.035	0.862	0.072	0.036	0.048
	*R*^2^ = 0.172, ∆*R*^2^ = 0.116, *F* = 45.974, *p* < 0.001			*R*^2^ = 0.208, ∆*R*^2^ = 0.163, *F* = 67.113, *p* < 0.001			*R*^2^ = 0.169, ∆*R*^2^ = 0.113, *F* = 45.138, *p* < 0.001		
Step 2	*β*	SE	*p*	*β*	SE	*p*	*β*	SE	*p*
Age	−0.040	0.052	0.440	−0.034	0.051	0.505	0.133	0.053	0.012
Sex	0.256	0.106	0.017	0.188	0.104	0.071	0.155	0.108	0.151
BMI	0.106	0.054	0.051	0.094	0.053	0.076	−0.032	0.055	0.564
Education	−0.015	0.033	0.656	0.032	0.033	0.322	0.100	0.034	0.004
Group	0.721	0.106	<0.000	0.852	0.104	<0.001	0.710	0.108	<0.001
	*R*^2^ = 0.240, ∆*R*^2^ = 0.068, *F* = 29.179, *p* < 0.001			*R*^2^ = 0.279, ∆*R*^2^ = 0.071, *F* = 32.292, *p* < 0.001			*R*^2^ = 0.202, ∆*R*^2^ = 0.033, *F* = 12.906, *p* < 0.001		
Step 3	*β*	SE	*p*	*β*	SE	*p*	*β*	SE	*p*
Age	−0.045	0.050	0.372	−0.039	0.049	0.428	0.130	0.052	0.012
Sex	0.219	0.102	0.033	0.150	0.099	0.132	0.127	0.106	0.233
BMI	0.092	0.052	0.076	0.080	0.051	0.113	−0.041	0.054	0.443
Education	−0.006	0.032	0.840	0.041	0.031	0.190	0.106	0.034	0.002
Group	0.549	0.107	<0.001	0.676	0.104	<0.001	0.594	0.111	<0.001
CTQ	0.279	0.052	<0.001	0.286	0.050	<0.001	0.192	0.053	<0.001

CTQ, childhood trauma questionnaire; DASS-21, Depression, Anxiety, Stress and Stress Scale-21; IES-R, Impact of Event Scale-Revised; PSQI, Pittsburgh sleep quality index; SE, standard error.

Spearman correlation analysis showed that the total scale of childhood trauma was positively related to psychological impact related to COVID-19 pandemic (*r* = 0.363, *p* < 0.001) and psychological distress (*r* = 0.330, *p* < 0.001) in schizophrenia, but the correlations between childhood trauma and global sleep quality (*r* = 0.133, *p* = 0.250), NGASR total score (*r* = 0.073, *p* = 0.250) were not statistically significant. The COVID-19 pandemic-related psychological impact was positively related to global sleep quality (*r* = 0.388, *p* < 0.001), psychological distress (*r* = 0.619, *p* < 0.001) and suicidal risk (*r* = 0.209, *p* = 0.015) in schizophrenia. The global sleep quality was significantly positive with psychological distress (*r* = 0.515, *p* < 0.001) and suicidal risk (*r* = 0.314, *p* < 0.001). Finally, psychological distress was positively correlated with suicidal risk (*r* = 0.303, *p* < 0.001) in patients with schizophrenia (Table 4).

**Table 4:** Bivariate Spearman correlations between main variables of interest in schizophrenia.

Variables of interest	1	2	3	4	5
CTQ total score	1.000				
IES-R total score	0.363***	1.000			
PSQI total score	0.133	0.388***	1.000		
DASS-21 total score	0.330***	0.619***	0.515***	1.000	
NGASR total score	0.073	0.209*	0.314***	0.303***	1.000

CTQ, childhood trauma questionnaire; DASS-21, Depression, Anxiety, Stress and Stress Scale-21; IES-R, Impact of Event Scale-Revised; NGASR, Nurses global assessment of suicide risk; PSQI, Pittsburgh sleep quality index. *p < 0.05, **p < 0.01, ***p < 0.001.

**Figure 2:**
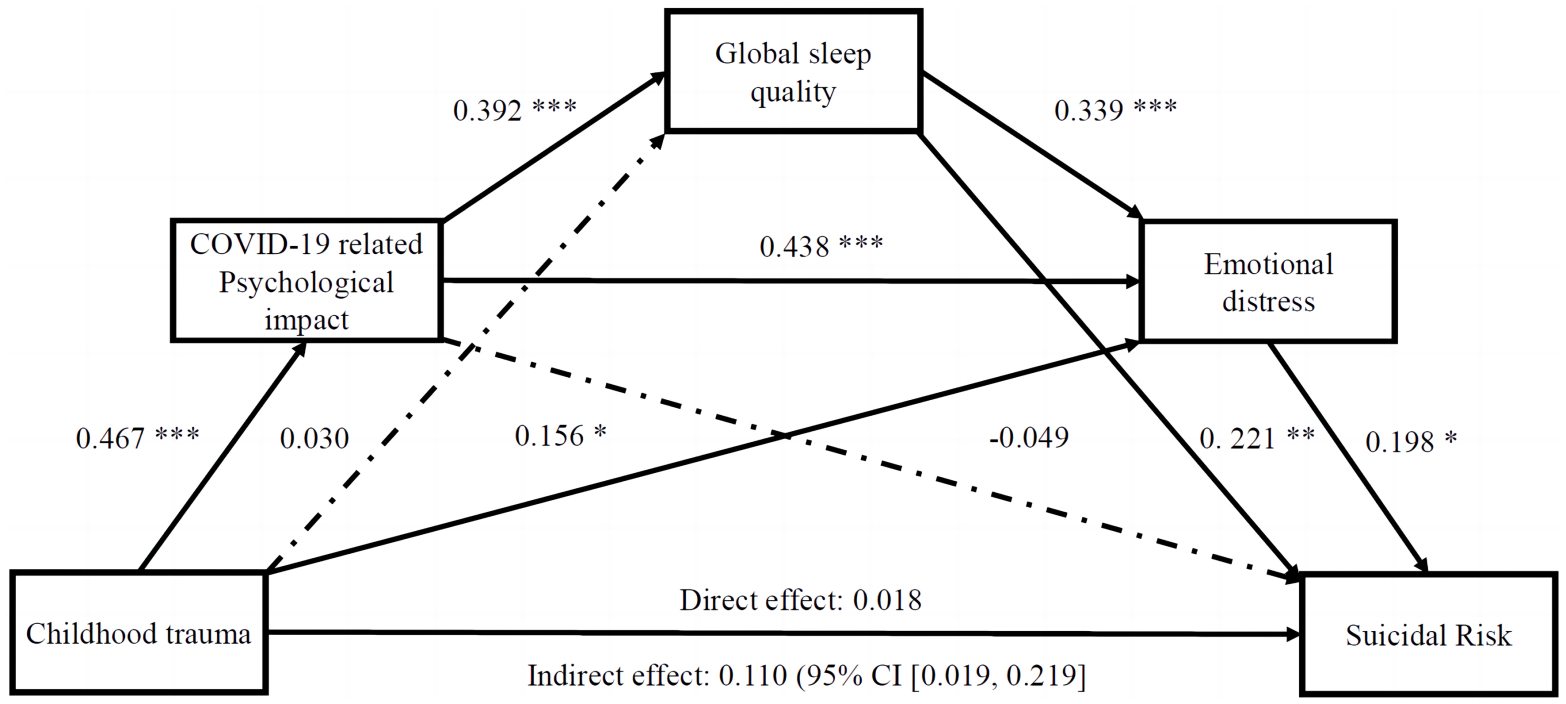
Tests of chain mediation model showed the sequential indirect effects of the perceived impact of the pandemic, global sleep quality and psychological distress in association between childhood trauma and suicidal risk in patients with schizophrenia. CTQ, childhood trauma questionnaire; DASS-21, Depression, Anxiety, Stress and Stress Scale-21; IES-R, Impact of Event Scale–Revised; PSQI, Pittsburgh Sleep Quality Index. *p < 0.05; **p < 0.01; ***p < 0.001.

Figure 2 presents the sequential mediated effects of psychological impact related to COVID-19, global sleep quality and psychological distress in association between childhood trauma and suicidal risk in schizophrenia. The relationship between childhood trauma and the psychological impact of COVID-19 was tested in the first model. The results (*R*^2^ = 0.205, *F* = 6.614, *p* < 0.001) indicate that childhood trauma is positively associated with the psychological impact of COVID-19 (*B* = 0.467, *p* < 0.001). Next, the model tests whether childhood trauma and psychological impact related to COVID-19 are directly associated with global sleep quality. The results (*R*^2^ = 0.190, *F* = 4.951, *p* < 0.001) indicate that the psychological impact of COVID-19 is directly associated with global sleep quality (*B* = 0.392, *p* < 0.001). However, childhood trauma was not directly associated with global sleep quality (*B* = 0.030, *p* = 0.308). The model next tests the relationship among childhood trauma, psychological impact of COVID-19, global sleep quality and psychological distress. The results (*R*^2^ = 0.524, *F* = 19.780, *p* < 0.001) show that childhood trauma (*B* = 0.156, *p* = 0.037), the psychological impact of COVID-19 (*B* = 0.438, *p* < 0.001) and global sleep quality (*B* = 0.339, *p* < 0.001) were directly and positively associated with psychological distress. Finally, we test the relationship among childhood trauma, psychological impact of COVID-19, global sleep quality, psychological distress and suicidal risk. The results (*R*^2^ = 0.251, *F* = 5.245, *p* < 0.001) show that global sleep quality (*B* = 0.221, *p* = 0.009) and psychological distress (*B* = 0.198, *p* = 0.049) were directly and positively associated with suicidal risk. But the childhood trauma (*B* = 0.018, *p* = 0.830) and psychological impact of COVID-19 (*B* = −0.049, *p* = 0.603) was not directly associated with suicidal risk (Supplementary Table S1). This study further conducted the bootstrapping method 5,000 times to test the significance of the mediating effect. The mediating effect of psychological impact related to pandemic and the sequential mediating effects of psychological impact and global sleep quality were significant if the 95% confidence interval did not include 0. However, the mediating effect of sleep quality was not significant if the 95% confidence interval included 0. The effect of childhood trauma on suicidal risk in schizophrenia was divided into a direct effect (*B* = 0.018, S.E. = 0.084, 95% CI −0.149/0.185) and total indirect effect (*B* = 0.110, S.E. = 0.051, 95% CI 0.019/0.219) (Supplementary Table S2). The effect of childhood trauma on suicidal risk in hospitalized schizophrenia patients was totally sequential mediated by the psychological impact of COVID-19, global sleep quality and psychological distress.

## 4. Discussion

In this study, there were three observations worth highlighting. First, consistent with previous studies, patients with schizophrenia experienced more childhood trauma and mental symptoms than HCs. Second, childhood trauma and a history of schizophrenia were associated with more traumatic symptoms related to COVID-19, poor sleep quality and severe depressive, anxiety and stress symptoms. Finally, the chain mediation model revealed that childhood trauma increased individuals’ stress response progressively during the COVID-19 pandemic, which triggered hyperarousal and led to poor sleep, further driving psychological distress and eventually increasing the suicidal risk for hospitalized patients with schizophrenia. Our findings may provide potential targets for the transformation of psychiatric care for patients with schizophrenia during the COVID-19 pandemic.

People with schizophrenia were significantly more likely to experience more childhood trauma and endorse poor sleep quality and severe traumatic symptoms related to COVID-19, depression, anxiety, and stress. Bolhuis et al. found that a higher genetic load of schizophrenia was associated with greater exposure to childhood trauma in the general pediatric population, which may partly explain why people with schizophrenia suffer more childhood trauma (39). One Mendelian randomization analysis found that there was a bidirectional causal relationship between childhood maltreatment and schizophrenia, indicating that childhood trauma increased the risk of schizophrenia and that patients with schizophrenia were more likely to experience more childhood trauma (40).

Our findings suggest that childhood trauma, regardless of a history of schizophrenia, was a good predictor for poor sleep quality and higher levels of traumatic symptoms, psychological distress. There are several potential mechanisms underlying childhood trauma and mental health. First, childhood trauma was associated with hyperarousal, which was caused by excessive activation of the hypothalamic–pituitary–adrenal (HPA) axis and sympathetic nervous system, and persistent alterations to neuroendocrine function contributed to poor sleep (41, 42). Second, previous studies have provided confirmed evidence that childhood trauma may sensitize people to future life stressors (24, 43). For example, individuals exposed to childhood trauma may have heightened psychological stress responses to COVID-19, thus leading to poor mental health. Third, van Nierop et al. propose an explanation that childhood trauma may not trigger specific mental illnesses, but may be associated with depression, psychosis, mania, and anxiety over the lifespan (23).

The current study revealed the mediation mechanism underlying the association between childhood trauma and suicidal risk in hospitalized patients with schizophrenia. Based on the chain mediation model, childhood trauma was positively associated with increased suicidal risk in schizophrenia. There was a framework referred to as “behavioral sensitization” that could partly explain the underlying mechanism. The concept stipulated that exposure to psychosocial stress (such as childhood trauma) may progressively increase the behavioral and biological response to subsequent exposures (44, 45). Our finding was consistent with previous studies showing that childhood trauma was positively associated with COVID-19-related psychological impact. The COVID-19 pandemic led to heightened psychological stress responses and the sensation of hyperarousal (44, 46). A review provided supportive evidence that hyperarousal processes, from the molecular to the higher system level, play key roles in the pathophysiology of insomnia (47). Moreover, insomnia was closely related to symptoms of depression, anxiety and stress (48). In addition, psychological distress, such as depression, anxiety or stress, might associate with increased suicidal risk among Chinese adults and patients with schizophrenia (49–52). This result adds to previous studies showing that childhood trauma was associated with increased suicidal risk with sequential mediation by the perceived impact of the pandemic, global sleep quality and psychological distress in hospitalized patients with schizophrenia. For example, patients with histories of childhood trauma exhibiting greater stress responses and hyperarousal during COVID-19 pandemic, accompanied by poor sleep quality and severe depression, anxiety and stress, eventually raised suicidal risk.

In fact, we should pay attention to the impact of childhood trauma and pandemic on the mental health of schizophrenia and propose more effective intervention measures to avoid relapse or aggravation of patients’ conditions. Trauma-informed practice, being familiar with the incidence of traumatic events and their impact on mental health, is one of the most effective approaches to avoid re-traumatization during psychiatric care. Applying principles of trauma-informed practice and humanizing psychiatric care, which may increase people’s sense of predictability and safety, improve therapeutic engagement, and support and help to buffer the immediate and long-term effects of psychological distress (53).

### 4.1. Limitations

There are several limitations worth noting in this study. First, the sample was not representative, which may be due to the single center and that all the samples were hospitalized patients in the acute stage. Second, the CTQ is a retrospective questionnaire, and this study cannot avoid recall bias. Third, this study only revealed correlational features due to its cross-sectional design. Longitudinal studies are needed to prove the direction or causal relationship between childhood trauma and psychological impacts related to COVID-19, sleep quality, psychological distress, and suicidal risk. Fourth, Fourth, the subject’s enrollment period was extensive. The psychological impact associated with the pandemic may change with the severity of the outbreak. Lastly, it should be noted that we did not evaluate suicide risk in the group of healthy controls, which prevented us from conducting a comparable mediating analysis in that population. For future investigations, it would be beneficial to incorporate a larger sample of healthy controls and investigate whether the potential mediating mechanism is consistent with that observed in hospitalized patients with schizophrenia.

## 5. Conclusion

Childhood trauma and schizophrenia could be good predictors for poor sleep, psychological impact related to COVID-19 and psychological distress. Moreover, the chain mediation analyses indicated a new underlying mechanism of the relationship between childhood trauma and suicidal risk in schizophrenia: childhood trauma was a good predictor for psychological distress via psychological impacts related to the COVID-19 pandemic, sleep quality and psychological distress. This study suggests a potential target to prevent the suicide risk in schizophrenia during a pandemic context.

## Data availability statement

The raw data supporting the conclusions of this article will be made available by the authors, without undue reservation.

## Ethics statement

The studies involving humans were approved by the Ethics Committee of the West China Hospital, Sichuan University. The studies were conducted in accordance with the local legislation and institutional requirements. Written informed consent for participation in this study was provided by the participants’ legal guardians/next of kin.

## Author contributions

MX, MZ, YX, LH, YT, JC, LZ, and YK participated and supervised data collection, and led the study protocol development and project administration. MX, XZ, MZ, and QW carried out the literature review and conceptualization. MX and XZ prepared the data for the analysis and wrote the main manuscript text. MX and XZ did the data analysis with advice from MZ and QW. YX, LH, YT, JC, LZ, and YK supported MX and XZ in the preparation of Figures 1, 2 and the interpretation of the results. MX, XZ, and MZ reviewed and edited the original draft. All authors reviewed the manuscript and contributed to its final draft.

## Footnotes

^1^ https://www.wjx.cn/app/survey.aspx
