# Supplementary material for: Childhood trauma and suicide risk in hospitalized patients with schizophrenia: the sequential mediating roles of pandemic related post-traumatic stress symptoms, sleep quality, and psychological distress
Source: Front Psychiatry. 2023 Sep 21;14:1221529. doi: 10.3389/fpsyt.2023.1221529 (PMC10551446; doi:10.3389/fpsyt.2023.1221529)
Supplement: Supplementary file 1 [file Table_1.docx]

Supplementary Material

Childhood trauma and suicide risk in hospitalized patients with schizophrenia: the sequential mediating roles of pandemic related post-traumatic stress symptoms, sleep quality, and psychological distress

**Min Xie ^1,2†^, Xuemin Zou ^1,2†^, Yingjing Xie ^1,2^, Li Hu ^1,2^, Yiguo Tang ^1,2^, Jia Cai ^1,2^, Yunxue Kuang ^3^, Ling Zhu ^4^, Min Zou ^5*^ and Qiang Wang ^1,2^* Correspondence:**Zou Min, Department of Pharmacy, West China Hospital, Sichuan University, Chengdu 610041, China.
[zoumin0818@scu.edu.cn](mailto:zoiumin0818@scu.edu.cn)

**Table S1. Results of mediation analysis. B = unstandardized coefficient (The continuous variables included in the mediation analysis are standardized).**

|  | Fit index | | |  |  |  |  | %95 CI | |
| --- | --- | --- | --- | --- | --- | --- | --- | --- | --- |
|  | R | R^2^ | F | B | SE | T | p | LLCI | ULCI |
| Dependent variable: IES-R | | | | | | | | | |
| CTQ | 0.453 | 0.205 | 6.614 | 0.467 | 0.085 | 5.492 | < 0.001 | 0.299 | 0.635 |
| Constant |  |  |  | 0.276 | 0.411 | 0.671 | 0.503 | -0.534 | 1.088 |
| Dependent variable: PSQI global sleep quality | | | | | | | | | |
| CTQ | 0.435 | 0.190 | 4.951 | 0.030 | 0.096 | 0.308 | 0.759 | -0.161 | 0.221 |
| IES-R |  |  |  | 0.392 | 0.090 | 4.343 | < 0.001 | 0.213 | 0.570 |
| Constant |  |  |  | -0.318 | 0.419 | 0.758 | 0.450 | -1.148 | 0.512 |
| Dependent variable: DASS-21 total score | | | | | | | | | |
| CTQ | 0.724 | 0.524 | 19.780 | 0.156 | 0.074 | 2.104 | 0.037 | 0.009 | 0.302 |
| IES-R |  |  |  | 0.438 | 0.074 | 5.910 | < 0.001 | 0.291 | 0.585 |
| PSQI |  |  |  | 0.339 | 0.068 | 4.983 | < 0.001 | 0.204 | 0.474 |
| Constant |  |  |  | 0.414 | 0.322 | 1.285 | 0.201 | -0.224 | 1.052 |
| Dependent variable: NGASR total score | | | | | | | | | |
| CTQ | 0.501 | 0.251 | 5.245 | 0.018 | 0.084 | 0.215 | 0.830 | 0.016 | 0.349 |
| IES-R |  |  |  | -0.049 | 0.094 | 0.521 | 0.603 | -0.235 | 0.137 |
| PSQI |  |  |  | 0.221 | 0.084 | 2.65 | 0.009 | 0.056 | 0.387 |
| DASS-21 |  |  |  | 0.198 | 0.100 | 1.984 | 0.049 | 0.001 | 0.396 |
| Constant |  |  |  | -0.158 | 0.364 | 0.433 | 0.666 | -0.878 | 0.563 |

BMI: body mass index; CTQ: childhood trauma questionnaire; DASS-21: Depression, Anxiety, Stress and Stress Scale-21; IES-R: Impact of Event Scale-Revised; NGASR: Nurses global assessment of suicide risk; PSQI: Pittsburgh sleep quality index; SE, standard error. The age, sex, BMI, and education were adjusted in all models.

**Table S2. Results of the chain mediating effect based on Bootstrapping Test.**

|  | Indirect effect | | | |
| --- | --- | --- | --- | --- |
|  | Beta | SE | LLCI | ULCI |
| Dependent variable: Nurses global assessment of suicide risk | | | | |
| CTQ → IES-R → Suicide risk | | | | |
|  | -0.023 | 0.045 | -0.120 | 0.057 |
| CTQ → IES-R → PSQI → Suicide risk | | | | |
|  | 0.041 | 0.021 | 0.012 | 0.102 |
| CTQ → IES-R → DASS-21 → Suicide risk | | | | |
|  | 0.041 | 0.030 | 0.001 | 0.114 |
| CTQ → IES-R → PSQI → DASS-21 → Suicide risk | | | | |
|  | 0.012 | 0.009 | 0.001 | 0.037 |
| CTQ → PSQI → Suicide risk | | | | |
|  | 0.007 | 0.024 | -0.035 | 0.064 |
| CTQ → PSQI → DASS-21 → Suicide risk | | | | |
|  | 0.002 | 0.008 | -0.012 | 0.021 |
| CTQ → DASS-21→ Suicide risk | | | | |
|  | 0.031 | 0.025 | -0.002 | 0.099 |
| Total mediation effects | | | | |
|  | 0.110 | 0.051 | 0.019 | 0.219 |
| Direct effect of childhood trauma on suicidal risk | | | | |
|  | 0.018 | 0.08 | -0.149 | 0.185 |

CTQ: childhood trauma questionnaire; DASS-21: Depression, Anxiety, Stress and Stress Scale-21; IES-R: Impact of Event Scale-Revised; NGASR: Nurses global assessment of suicide risk; PSQI: Pittsburgh sleep quality index; Beta: standardized coefficient; SE: standard error; LLCI: lower level of confidence interval; ULCI: upper level of confidence interval.
